# Evaluation of a New ^177^Lu-Labeled Somatostatin Analog for the Treatment of Tumors Expressing Somatostatin Receptor Subtypes 2 and 5

**DOI:** 10.3390/molecules25184155

**Published:** 2020-09-11

**Authors:** Rosalba Mansi, Guillaume Pierre Nicolas, Luigi Del Pozzo, Karim Alexandre Abid, Eric Grouzmann, Melpomeni Fani

**Affiliations:** 1 Division of Radiopharmaceutical Chemistry, Clinic of Radiology and Nuclear Medicine, University Hospital Basel, 4031 Basel, Switzerland; rosalba.mansi@usb.ch (R.M.); Luigi.DelPozzo@usb.ch (L.D.P.); 2 Division of Nuclear Medicine, Clinic of Radiology and Nuclear Medicine, University Hospital Basel, 4031 Basel, Switzerland; guillaume.nicolas@usb.ch; 3 Catecholamine and Peptides Laboratory, Department of Laboratories, University Hospital of Lausanne, 1011 Lausanne, Switzerland; Karim-Alexandre.Abid@chuv.ch (K.A.A.); eric.grouzmann@chuv.ch (E.G.)

**Keywords:** radiolabeled somatostatin analogs, SST2, SST5, Lu-177, targeted radionuclide therapy, neuroendocrine tumors, dual-tumor mouse model

## Abstract

Targeted radionuclide therapy of somatostatin receptor (SST)-expressing tumors is only partially addressed by the established somatostatin analogs having an affinity for the SST subtype 2 (SST2). Aiming to target a broader spectrum of tumors, we evaluated the bis-iodo-substituted somatostatin analog ST8950 ((4-amino-3-iodo)-d-Phe-c[Cys-(3-iodo)-Tyr-d-Trp-Lys-Val-Cys]-Thr-NH_2_), having subnanomolar affinity for SST2 and SST5, labeled with [^177^Lu]Lu^3+^ via the chelator DOTA (1,4,7,10-tetraazacyclododecane-1,4,7,10-tetraacetic acid). Human Embryonic Kidney (HEK) cells stably transfected with the human SST2 (HEK-SST2) and SST5 (HEK-SST5) were used for in vitro and in vivo evaluation on a dual SST2- and SST5-expressing xenografted mouse model. ^nat^Lu-DOTA-ST8950 showed nanomolar affinity for both subtypes (IC_50_ (95% confidence interval): 0.37 (0.22–0.65) nM for SST2 and 3.4 (2.3–5.2) for SST5). The biodistribution of [^177^Lu]Lu-DOTA-ST8950 was influenced by the injected mass, with 100 pmol demonstrating lower background activity than 10 pmol. [^177^Lu]Lu-DOTA-ST8950 reached its maximal uptake on SST2- and SST5-tumors at 1 h p.i. (14.17 ± 1.78 and 1.78 ± 0.35%IA/g, respectively), remaining unchanged 4 h p.i., with a mean residence time of 8.6 and 0.79 h, respectively. Overall, [^177^Lu]Lu-DOTA-ST8950 targets SST2-, SST5-expressing tumors in vivo to a lower extent, and has an effective dose similar to clinically used radiolabeled somatostatin analogs. Its main drawbacks are the low uptake in SST5-tumors and the persistent kidney uptake.

## 1. Introduction

Targeted radionuclide therapy of neuroendocrine tumors (NET) via somatostatin receptor (SST) is proven very effective. The NETTER-1 phase III study showed that treatment with the ^177^Lu-labeled somatostatin analog DOTA-TATE ([1,4,7,10-tetraazacyclododecane-1,4,7,10-tetraacetic acid^0^, Tyr^3^,Thr^8^]-octreotate), in combination with single-dose nonradiolabeled octreotide (Sandostatin^®^ LAR^®^), significantly improved objective response, progression-free survival and quality of life versus treatment with double-dose octreotide [1,2]. Earlier studies involving large cohorts treated with the ^90^Y- or ^177^Lu-labeled analog DOTA-TOC ([DOTA^0^, Tyr^3^]-octreotide) support these findings [3,4]. [^177^Lu]Lu-DOTA-TOC is under evaluation in the phase III trial COMPETE (NCT03049189) versus the mTOR inhibitor, everolimus, while [^177^Lu]Lu-DOTA-TATE (Lutathera^®^) is approved by the U.S. Food and Drug Administration (FDA) and the European Medicines Agency (EMA). As a companion to these analogs for radionuclide therapy, two ^68^Ga-labeled radio-diagnostics, [^68^Ga]Ga-DOTA-TATE (NETSPOT^®^) and [^68^Ga]Ga-DOTA-TOC (SOMAKIT TOC^®^), received FDA approval for positron emission tomography (PET) imaging. The molecular basis of these successful approvals rests on the high affinity of the (radio)metallated DOTA-TATE and DOTA-TOC for the somatostatin receptor subtype 2 (SST2), which is known to be overexpressed by NET cells. However, poorly differentiated neuroendocrine carcinoma (NEC), high-grade NETs, and to a certain extent, well-differentiated NETs, may show low and/or heterogeneous SST2 expression [5,6], leading to suboptimal tumor targeting with these analogs. On the other hand, these tumors may express or co-express other SST subtypes among the five known ones (SST1-5). SST5 is the second highly expressed subtype in gastroenteropancreatic neuroendocrine tumors (GEP-NETs) [7], behind the predominant expression of SST2 (at least in primary tumors) and is concomitantly expressed with SST2 in 70–100% of GEP-NETs, in breast cancer, growth hormone (GH)-secreting pituitary adenomas and in 20–50% of intestinal or bronchial NETs [5,8,9,10]. NETs from G1/2 to G3 show a downregulation of SST2, while SST5 is constantly present [5,11]. SST5 is predominantly expressed, compared to SST2, in other tumors such as glioblastomas [12], tumor capillaries of pancreatic adenocarcinomas [13] or in lung cancer [14]. There are also cases of NETs where SST2 is absent while SST5 is present [6,15]. There are other tumors where SST5 is expressed, while SST2 is absent, including ACTH pituitary adenoma, cervix carcinoma and ovarian carcinoma [16]. Therefore, analogs targeting SST2 and SST5 (but also other subtypes), may potentially target a broader spectrum of various tumors and/or increase the radiation dose in a given tumor.

Most of the known analogs are synthetic cyclic octapeptides with a disulfide six-membered ring and SST2-selectivity. We are interested in developing radiolabeled somatostatin analogs for multireceptor subtype targeting. Replacement of key amino acids on the octreotide motif resulted in the analog [DOTA, 1-Nal^3^]-octreotide (DOTA-NOC) with a high affinity for the SST2 and SST5 and lower for the SST3 [17]. Other designs include, highly constrained bicyclic octreotide analogs, consisting of a head-to-tail cyclization and an inner disulfide six-membered ring [18], the head-to-tail cyclo-hexapeptide pasireotide (Signifor or SOM230) [19], cyclic nonapeptides with nondisulfide eight-membered ring [20], and the 14mer and pseudo-14mer cyclic somatostatin-14 (SS-14) mimics, with ring-size of 12, 9, 8 and 6 amino acids—with the higher number ring favoring multireceptor subtype recognition [21]. All the above-mentioned analogs showed certain limitations, with [^68^Ga]Ga-DOTA-NOC being, so far, the only well-established analog for SST2, SST3 and SST5 targeting [22]. Clinical data with [^68^Ga]Ga-DOTA-NOC indicate that multi-receptor subtype targeting is relevant for improving the diagnostic accuracy and sensitivity of PET imaging of SST-expressing tumors [23,24]. Such clinical data on therapy are lacking. In fact, the therapeutic equivalent [^177^Lu]Lu-DOTA-NOC has been evaluated in only 69 NET patients, compared to [^177^Lu]Lu-DOTA-TATE, showing higher uptake in normal tissues with subsequently higher effective dose [25]. None of the other analogs has been evaluated for targeted radionuclide therapy.

In this work, we evaluate the ^177^Lu-labeled somatostatin analog ST8950 ((4-amino-3-iodo)-d-Phe-c[Cys-(3-iodo)-Tyr-d-Trp-Lys-Val-Cys]-Thr-NH_2_) for potential treatment of SST2- and SST5-expressing tumors. ST8950 (identified as peptide #9 in [26] and as AP102 in [27,28] is a disulfide-bridged bis-iodo-substituted octapeptide that exhibits sub-nanomolar affinity to SST2 and SST5 [26]. ST8950 is as potent as the natural SS-14 in its ability to inhibit growth hormone and prolactin release [26], it has intermediate agonistic potency between octreotide and pasireotide at both subtypes [27], and it acutely reduces growth hormone secretion without causing hyperglycemia (a known undesirable effect of pasireotide) in a healthy rat model [28]. Our previous work demonstrated that coupling of the chelator DOTA and complexation of Ga^3+^ does not alter the affinity to SST2, and while it reduces its affinity to SST5, this still remains in a low nanomolar range [29]. [^68^Ga]Ga-DOTA-ST8950 showed high and specific accumulation in SST2 and SST5-expressing tumors in vivo, comparable to [^68^Ga]Ga-DOTA-NOC [29]. Herein we report the comprehensive evaluation of the therapeutic equivalent [^177^Lu]Lu-DOTA-ST8950. The influence of Lu-complexation on the affinity for SST2 and SST5 was assessed, together with the internalization and efflux rate of [^177^Lu]Lu-DOTA-ST8950 vs. [^177^Lu]Lu-DOTA-NOC on intact cells. A series of in vivo characteristics, including the influence of the injected peptide masses on the biodistribution, the specificity, the role of nephroprotective agents on the kidney uptake, the pharmacokinetics over 168 h, the residence time on the tumors and critical organs and finally the dosimetry of [^177^Lu]Lu-DOTA-ST8950 were assessed on a dual SST2- and SST5-expressing xenografted model.

## 2. Results and Discussion

### 2.1. Synthesis of the (Radio)Metallated Peptide Conjugates, Stability and Lipophilicity

**Figure 1:**
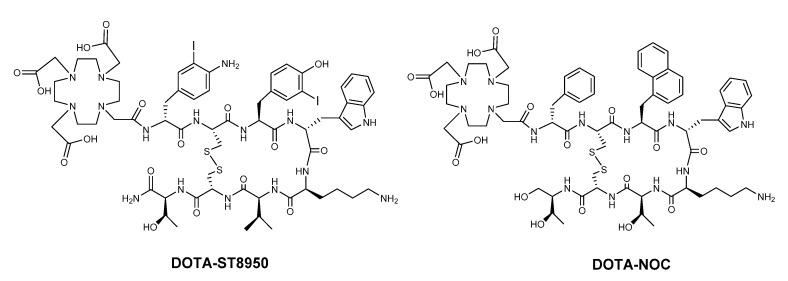
The structural formulae of DOTA-ST8950 (DOTA-(4-amino-3-iodo)-d-Phe-c[Cys-(3-iodo)-Tyr-d-Trp-Lys-Val-Cys]-Thr-NH_2_) and DOTA-NOC (DOTA-d-Phe-c[Cys-1-NaI-d-Trp-Lys-Thr-Cys]Thr(ol)).

The ^nat^Lu complexes of DOTA-ST8950 and DOTA-NOC (Figure 1) were obtained with a yield of 70–80%, based on the initial amount of peptide used.

The purity and identity were confirmed by reverse-phase high-performance liquid chromatography (RP-HPLC) and electrospray ionization mass spectrometry (ESI-MS). The analytical data of the peptide conjugates and their ^nat^Lu complexes are reported in Table 1.

^177^Lu-labeling did not require any purification step. Radiochemical yield (non-isolated product, estimated by radio-HPLC) was > 95% and radiochemical purity > 93%. The radiochemical purity of [^177^Lu]Lu-DOTA-ST8950 without any formulation or addition of scavengers was decreased over a period of 24 h to 74%, when the radiotracer was stored at 4 °C, and to 67% when it was stored at room temperature (RT).

[^177^Lu]Lu-DOTA-ST8950 was found to be more lipophilic than the reference compound [^177^Lu]Lu-DOTA-NOC, with log *D* = −1.2 ± 0.1 vs. −1.6 ± 0.1, respectively.

### 2.2. Binding Affinity Studies

The results of the binding affinity for the human SST2 and SST5 are summarized in Table 2. Conjugation of the chelate ^nat^Lu-DOTA to ST8950 did not alter its binding affinity to SST2 (IC_50_: 0.37 vs. 0.28 nM for ^nat^Lu-DOTA-ST8950 and ST8950, respectively) but reduced by more than a factor of four its affinity to SST5 (IC_50_: 3.4 vs. 0.77 nM, respectively). This observation is in agreement with the affinity data of ^nat^Ga-DOTA-ST8950 having the same affinity as ST8950 for SST2, but reduced affinity for SST5 [29]. The loss of affinity for SST5 after chelate conjugation is more prominent with ^nat^Lu-DOTA (factor of four) than with ^nat^Ga-DOTA (factor of two); IC_50_: 3.4 nM for ^nat^Lu-DOTA-ST8950 vs. 1.9 nM for ^nat^Ga-DOTA-ST8950 vs. 0.77 nM for ST8950.

The affinity of ^nat^Lu-DOTA-ST8950 was compared favorably with ^nat^Lu-DOTA-NOC in both receptor subtypes (IC_50_: 0.37 vs. 0.51 nM in SST2 and 3.4 vs. 4.8 nM in SST5, respectively). When compared with the native hormone SS-14, ^nat^Lu-DOTA-ST8950 and ^nat^Lu-DOTA-NOC had slightly lower affinities for the SST2 and significantly lower for the SST5.

Overall, the conjugation of a chelate affects the affinity of ST8950 to SST5, but not to SST2, with the ^nat^Lu-DOTA-ST8950 having lower affinity than its ^nat^Ga-equivalent. This is in agreement with the observation made previously by Antunes et al. [30] regarding the lower affinity of ^nat^Lu-DOTA-NOC, compared to its ^nat^Ga-equivalent, which was attributed to the different coordination number and geometry between the ^nat^Ga and ^nat^Lu complexes.

### 2.3. In Vitro Characterization

#### 2.3.1. Internalization Studies

**Figure 2:**
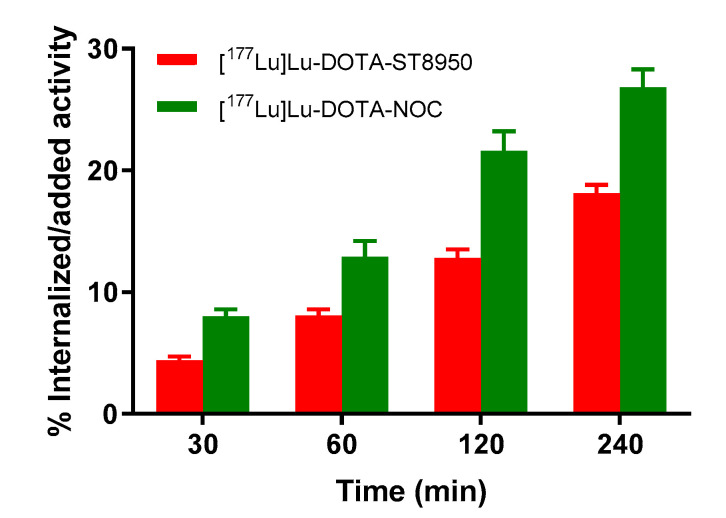
Internalization of [^177^Lu]Lu-DOTA-ST8950 and [^177^Lu]Lu-DOTA-NOC in Human Embryonic Kidney (HEK)-SST2 at 37 °C. The results are expressed as % (mean ± SD) of applied activity in the cells and normalized per million cells. All values refer to specific internalization after subtracting the nonspecific (measured in the presence of 1000-fold excess of SS-14) from the total internalized fraction, at each time point.

The internalization rate in Human Embryonic Kidney (HEK)-somatostatin receptor subtype 2 (SST2) cells is reported in Figure 2. [^177^Lu]Lu-DOTA-ST8950 and [^177^Lu]Lu-DOTA-NOC showed specific and time-dependent cellular uptake. [^177^Lu]Lu-DOTA-ST8950 showed lower internalization compared with [^177^Lu]Lu-DOTA-NOC (18.1 ± 0.7 vs. 26.8 ± 0.1% at 4 h, respectively). The percentage of the cell-surface-bound fraction was very low (about 1%) in all cases, demonstrating that the cell-surface-bound radiotracer is rapidly internalized inside the cells. This confirms their agonistic nature.

Neither of the two radiotracers had substantial internalization on HEK-SST5 cells (0.5–1.2%, at 4 h). The lack of internalization on HEK-SST5 cells of radiolabeled analogs with an affinity for this subtype, like [^177^Lu]Lu-DOTA-ST8950 and [^177^Lu]Lu-DOTA-NOC, has been observed by others [30,31], while we and others confirmed these results specifically for [^67^Ga]Ga-DOTA-NOC [29,30] and for [^67^Ga]Ga-DOTA-ST8950 [29]. Despite the lack of internalization in vitro, these analogs are able to bind to SST5-expressing tumors in vivo [29].

Unfortunately, no in vitro data are available for [^177^Lu]Lu-DOTA-NOC to allow direct comparison. Nevertheless, our internalization results on [^177^Lu]Lu-DOTA-NOC are in agreement with the data reported on [^111^In]In-DOTA-NOC in HEK-SST2 [30]. This is also in line with the similar affinities found for ^nat^Lu-DOTA-NOC and ^nat^In-DOTA-NOC, measured in the same assay [30].

#### 2.3.2. Efflux Studies

**Figure 3:**
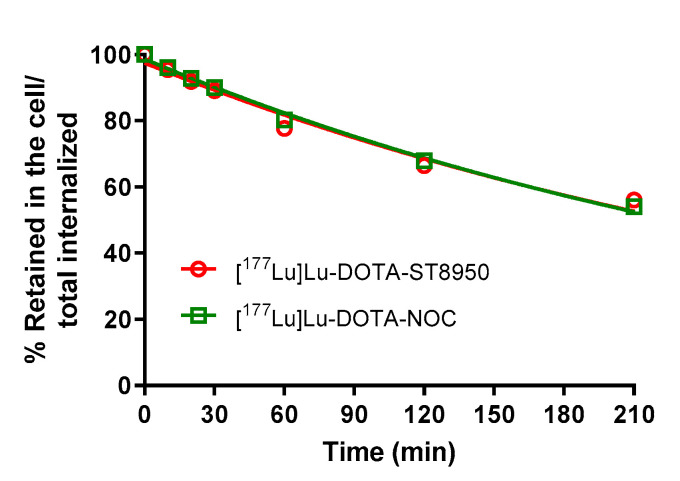
Cellular retention of [^177^Lu]Lu-DOTA-ST8950 and [^177^Lu]Lu-DOTA-NOC in HEK-SST2 after 2 h of incubation at 37 °C and acid wash to remove the cell-surface-bound fraction. The results are expressed as % (mean ± SD) of the internalized activity.

The cellular retention of [^177^Lu]Lu-DOTA-ST8950 and [^177^Lu]Lu-DOTA-NOC in HEK-SST2 is presented in Figure 3. Both radiotracers showed the same efflux rate, with more than 50% remaining inside the cells (internalized) after 4 h at 37 °C.

### 2.4. In Vivo Evaluation of [^177^Lu]Lu-DOTA-ST8950 in Tumor-Bearing Mice

#### 2.4.1. Influence of the Injected Peptide Mass

Biodistribution studies at two different peptide masses, 10 pmol and 100 pmol, were performed at 1 and 4 h p.i. to determine whether the amount of the injected peptide influences the distribution of [^177^Lu]Lu-DOTA-ST8950 and its uptake in the tumors and organs. The results are reported in Table 3 and refer to a dual SST2- and SST5-tumor mouse model.

[^177^Lu]Lu-DOTA-ST8950 performed better when injected in higher amounts (100 vs. 10 pmol), as this led to a more desirable lower uptake in the blood, in the blood-rich organs, such as the spleen, lungs, bone marrow, and in other organs, such as the intestine and stomach. The uptake in SST2- and SST5-expressing tumors remained unchanged at 1 h p.i., but it was greater for the higher peptide mass on SST2-tumor at 4 h p.i. For all other organs, the uptake was at the same level for both peptide masses.

The “suppressed” uptake by increasing the injected peptide mass in certain organs expressing SST and in the blood resulted in improved tumor-to-background ratios. This has been reported in the literature [32] and we hypothesize that it is partially attributed to receptor saturation in vivo. Even though the binding of [^177^Lu]Lu-DOTA-ST8950 was only tested for the human SST2 and SST5, which are expressed on the tumors, we contemplate that this is very similar to the mouse SST2 and SST5, which are expressed in certain organs through the mouse body. This is because the somatostatin receptors are highly conserved with 82–99% amino acid homology between humans and rodents, depending on the subtype [33,34,35]. More specifically, 93–96% sequence identity between the human, rat, mouse, porcine and bovine SST2 subtype and 82–83% sequence homology between human and rodent SST5. The fact that tumor uptake was not reduced might be explained by a higher receptor density in the tumors, compared to non-tumor-bearing organs, where only further increases in the injected peptide mass can produce saturation. Knowing the effect of the peptide mass is essential when therapy is being planned as it may reduce the radiation exposure of certain non-targeted organs and possibly whole-body radiation dose, without influencing the tumor dose.

The tumor uptake of [^177^Lu]Lu-DOTA-ST8950 was found to be 1.8 (14.17 ± 1.78 vs. 26.27 ± 8.20%IA/g, *p* = 0.0084) and 8.3 (1.78 ± 0.35 vs. 14.87 ± 5.90%IA/g, *p* = 0.0005) times lower in SST2- and SST5-expressing tumors, respectively, compared with [^68^Ga]Ga-DOTA-ST8950 at the same time point (1 h p.i.) and with the same peptide mass (100 pmol) [29]. Unfortunately, ex vivo histology to confirm the in situ expression of SST2 and SST5 and compare it with the expression on the [^68^Ga]Ga-DOTA-ST8950 study was not feasible. Nevertheless, the SST2 and SST5 expression on the HEK transfected cells used on the xenograft model had been confirmed by western blot [27]. On the other hand, [^177^Lu]Lu-DOTA-ST8950 had significantly lower uptake in the blood (0.96 ± 0.23 vs. 1.86 ± 0.56%IA/g, *p* = 0.001), the liver (2.60 ± 0.42 vs. 6.39 ± 1.93%IA/g, *p* = 0.0001) and the kidneys (9.88 ± 0.99 vs. 14.13 ± 3.69%IA/g, *p* = 0.0095), compared with [^68^Ga]Ga-DOTA-ST8950, leading to better or similar tumor-to-non tumor ratios, based on the SST2-tumor uptake.

The discordance between ^177^Lu-labeled and ^68^Ga-labeled conjugates, such as [^68^Ga]Ga-/[^177^Lu]Lu-DOTA-ST8950, needs to be considered for all theranostic pairs. This affects for example dosimetry studies performed with the diagnostic companion for therapy planning. It is known that tumor-targeting properties, pharmacokinetics and body distribution of radiotracers may be affected by selecting a different radiometal. Therefore, each radiotracer needs to be evaluated independently and modifications implemented accordingly to produce a “matching” pair. There are examples in the literature where the theranostic pair consists of two radiotracers that differ not only by the radiometal, but also by the conjugate, e.g., [^68^Ga]Ga-OPS202/[^177^Lu]Lu-OPS201 ([^68^Ga]Ga-NODAGA-JR11/[^177^Lu]Lu-DOTA-JR11) for SST2 [36] or ^68^Ga-pentixafor/^177^Lu-pentixather for CXCR4 [37].

#### 2.4.2. Metabolic Stability

In vivo metabolic stability of [^177^Lu]Lu-DOTA-ST8950 was assessed by radio-HPLC on blood samples of mice collected at 30 and 60 min after injection of the radiotracer. [^177^Lu]Lu-DOTA-ST8950 showed high in vivo stability, with 90% remaining intact in the blood 60 min after injection.

#### 2.4.3. In Vivo Specificity and Kidney Protection

The uptake of [^177^Lu]Lu-DOTA-ST8950 in SST-negative tumors (Table 4) was very low (0.70 ± 0.08 vs. 15.50 ± 3.63%IA/g, *p* < 0.0001 vs. 1.87 ± 0.49%IA/g, *p* = 0.0014, in SST2- and SST5-tumors, respectively), confirming the in vivo receptor-mediated uptake (specificity).

The pre-injection of the lysine reduced the uptake of the radiotracer in the kidneys by 40% (from 9.75 ± 1.87 to 5.93 ± 0.56%IA/g, *p* < 0.0001), without influencing the total-body biodistribution and the uptake in the tumors (Table 4).

The results indicate that the cationic amino acids such as lysine and arginine that are used for kidney protection in neuroendocrine tumor patients treated with [^177^Lu]Lu-DOTA-TATE or [^177^Lu]Lu-DOTA-TOC [38] can also be used in combination with [^177^Lu]Lu-DOTA-ST8950, with similar effects.

#### 2.4.4. Pharmacokinetics of [^177^Lu]Lu-DOTA-ST8950

The biodistribution of [^177^Lu]Lu-DOTA-ST8950 was studied at 1, 4, 48, 72 and 168 h p.i. The results are presented in Table 5. [^177^Lu]Lu-DOTA-ST8950 was predominantly accumulated in the SST2-expressing tumors, while its accumulation in the SST5-expressing tumors was significantly lower. Normal distribution was also seen in the SST-expressing organs, such as the pancreas, stomach and adrenals. The maximum tumor uptake was observed already at 1 h p.i. remaining essentially unchanged at 4 h p.i. (SST2-tumor: 14.17 ± 1.78 and 15.50 ± 3.63%IA/g (*p* = 0.459), respectively and SST5-tumor: 1.78 ± 0.35 and 1.87 ± 0.49%IA/g (*p* = 0.483), respectively).

The radiotracer was cleared rapidly from the blood; only 0.02 %IA/g remained in the blood at 24 h p.i. The kidney was the second organ accumulating activity after the SST2-tumors. The kidney uptake was high (approx. 10%IA/g) at the initial time points of the study, i.e., 1 and 4 h p.i., and was washed out over time, without, however, being negligible 168 h later (1.42 ± 0.38%IA/g). Lung and liver also showed considerable uptake. The uptake in the lungs was washed out quickly, while the uptake in the liver was more persistent and mainly attributed to the lipophilicity of the radiotracer.

**Figure 4:**
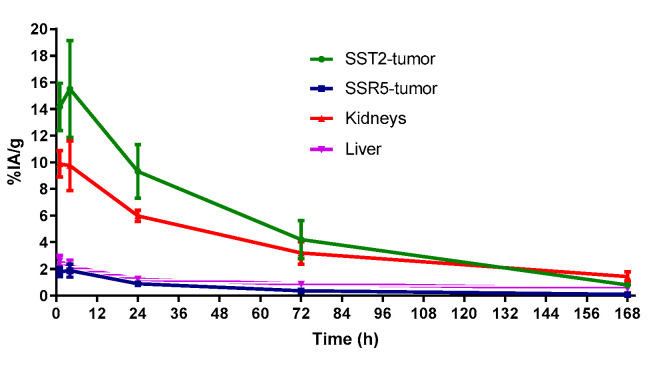
The area under the time–activity curve (AUC) in the SST2- and SST5-tumors, in the kidneys and the liver. These pharmacokinetic data were generated from serial independent biodistribution experiments performed 1, 4, 24, 72 and 168 h post injection.

Figure 4 shows the area under the time–activity curve (AUC) in SST2- and SST5-tumors and also in the liver and kidneys. The mean residence time for the SST2-tumor was 8.6 h, for the SST5-tumor 0.79 h, for the kidneys 6.3 h and for the liver 1.6 h, based on nondecay corrected biodistribution data and normalized per gram of tumor.

The low accumulation in SST5-tumors is in contrast with the uptake of the [^68^Ga]Ga-DOTA-ST8950 in the same tumor model. Unfortunately, very limited biodistribution data of somatostatin analogs in SST5-expressing tumors are available for comparison and a better understanding of these findings. One such case is the pan-somatostatin analog [^111^In]In-DOTA-LLT-SS28 [39]. [^111^In]In-DOTA-LLT-SS28 showed 1.7 times higher accumulation in SST5-tumors (2.61 ± 0.39 vs. 1.52 ± 0.75 %IA/g, respectively), when compared with [^177^Lu]Lu-DOTA-ST8950 in the same animal model and experimental conditions (4 h after injection of 10 pmol of the radiotracer). However, [^111^In]In-DOTA-LLT-SS28 had 2.3 times lower uptake in the SST2-tumor than [^177^Lu]Lu-DOTA-ST8950 (4.43 ± 1.52 vs. 10.34 ± 2.78%IA/g, respectively) under the same conditions. These data are in agreement with the higher affinity of ^nat^Lu-DOTA-ST8950 vs. ^nat^In-DOTA-LLT-SS28 for SST2 (IC_50_ = 0.35 vs. 1.8 nM) and the lower affinity for SST5 (IC_50_ = 3.4 vs. 1.4 nM) [39].

Overall, the biodistribution profile and pharmacokinetics of [^177^Lu]Lu-DOTA-ST8950 follow the profile of known radiolabeled somatostatin analogs, nevertheless, its high and persistent accumulation in the kidneys is identified as the main drawback for targeted radionuclide therapy.

#### 2.4.5. SPECT/CT Imaging

**Figure 5:**
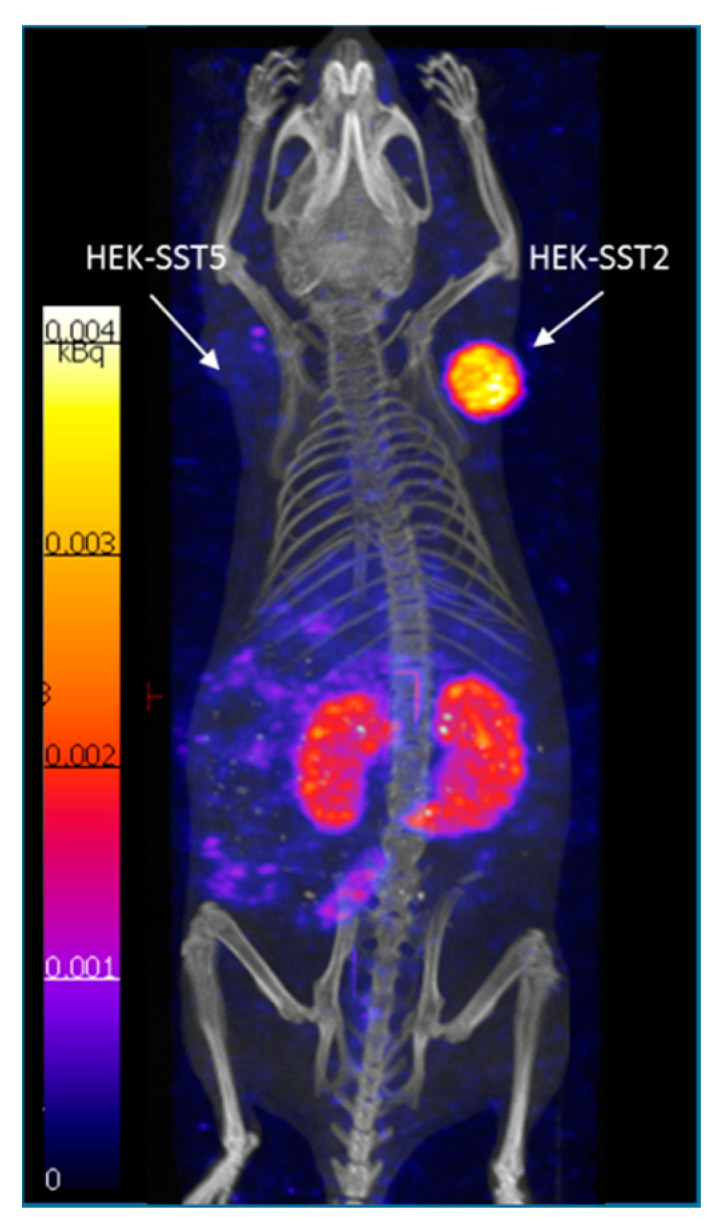
Maximum intensity projection (MIP) SPECT/CT images of [^177^Lu]Lu-DOTA-ST8950 in a dual SST2- and SST5-expressing tumor mouse model, 4 h after injection.

SPECT/CT image 4 h after injection of [^177^Lu]Lu-DOTA-ST8950 is shown in Figure 5. [^177^Lu]Lu-DOTA-ST8950 clearly visualized the SST2-expressing tumors, but had faint uptake on the SST5 tumors, which confirm the low uptake in SST5 xenografts in the biodistribution studies. The background activity of [^177^Lu]Lu-DOTA-ST8950 was low at 4 h after injection, with abdominal uptake and kidney uptake indicative of its renal excretion.

#### 2.4.6. Radiation Dosimetry Data Extrapolated to Humans

Table 6 shows the radiation dose estimate for human organs and tumors after injection of 100 pmol [^177^Lu]Lu-DOTA-ST8950, based on a female phantom. The estimated whole-body radiation dose (effective dose) of [^177^Lu]Lu-DOTA-ST8950 was 0.0252 mSv/MBq, which is within the expected range for ^177^Lu-labeled somatostatin analogs. The radiation dose delivered to SST2-tumors was higher by a factor of seven than the dose delivered to SST5-tumors. A higher radiation dose targeting the SST5-expressing tumors is desirable to produce effective treatment outcomes. However, in many cases where SST5 is co-expressed with SST2, such as the indications mentioned in the introduction, this dual-targeting brings additive value, even if the uptake on SST5 is not at the same level as on SST2.

Comparison between [^177^Lu]Lu-DOTA-ST8950 and the FDA approved [^177^Lu]Lu-DOTA-TATE is not easy since the results of [^177^Lu]Lu-DOTA-TATE refer to an injected peptide mass of 10 pmol [32], though calculated with the same methodology. However, the following comparison can be made: (a) Given that the renal uptake and the kinetic of washout are not affected by the injected amount of peptide, the comparison between the two radiotracers is valid. [^177^Lu]Lu-DOTA-ST8950 delivers a 2.5-fold higher radiation dose to the kidneys than [^177^Lu]Lu-DOTA-TATE. This is a potential limitation, due to the known nephrotoxicity of this type of treatment. Unfortunately, the higher [^177^Lu]Lu-DOTA-ST8950 uptake in the kidneys, compared to [^177^Lu]Lu-DOTA-TATE, persists even with the use of basic amino acids that enable kidney uptake reduction by 40% with both radiolabeled analogs. On the other hand, SST5 co-targeting increases the radiation dose to the tumor and enhances the therapeutic effect, compared to SST2-selective targeting. This might mitigate the additional kidney dose by balancing the tumor-to-kidney ratio. (b) Regarding hematotoxicity, the other limiting factor in this type of treatment, the red marrow dosimetry of [^177^Lu]Lu-DOTA-ST8950 is at about the same level as with [^177^Lu]Lu-DOTA-TATE, therefore, there are no additional concerns than what is already known. (c) Last, but not least, [^177^Lu]Lu-DOTA-ST8950 delivers a much higher radiation dose to the liver than [^177^Lu]Lu-DOTA-TATE, presumably as a consequence of its lipophilicity. Nevertheless, the liver uptake is of greater concern for the diagnostic radiotracer [^68^Ga]Ga-DOTA-ST8950 considering that the liver is the first site of metastasis of NETs and low background activity is needed for good image contrast and diagnostic accuracy.

## 3. Materials and Methods

All chemicals were obtained from commercial sources and used without additional purification. ESI-MS was carried out with ESI Bruker Esquire 3000 plus (Bruker Daltonics, Billerica, MA, USA). RP-HPLC was performed on a Bischoff instrument consisting of a LC-CaDi 22–14 interface, a UV-vis Lambda 1010 detector and a flow-through Berthold LB509 γ-detector (BISCHOFF chromatography, Leonberg, Germany), using a Phenomenex Jupiter Proteo 90 Å C12 (250 × 4.6 mm) column (Phenomenex Inc., California, USA). (Eluents: A = H_2_O (0.1% TFA), B = Acetonitrile (0.1% TFA), Gradient: 95–50% solvent A in 15 min, Flow rate: 1.5 mL/min). Quantitative gamma counting was performed on a COBRA 5003 γ-system well counter from Packard Instruments (Meriden, CT, USA). SPECT/CT images were acquired using a dedicated nanoSPECT/CT system (Bioscan, Mediso, Budapest, Hungary).

Human Embryonic Kidney (HEK) cells were stably transfected with plasmids encoding the human SST2 and SST5 (HEK-SST2 and HEK-SST5) and cultivated as previously described [27]. The SST2 and SST5 expression was confirmed by western blot and has been previously reported [27]. Nontransfected HEK cells were used as negative control.

### 3.1. Synthesis of the (Radio)Metallated Peptide Conjugates and Stability

DOTA-ST8950 (Figure 1) was custom-made by PolyPeptide (San Diego, CA, USA). DOTA-NOC (Figure 1) was synthesized by standard Fmoc-solid-phase peptide synthesis, purified by preparative RP-HPLC and characterized by ESI-MS and analytical RP-HPLC (Figure 1). ^nat^Lu-DOTA-ST8950 and ^nat^Lu-DOTA-NOC were synthesized after incubation of the DOTA-conjugates with a 2.5-fold excess of ^nat^LuCl_3_ × 6H_2_O (Sigma Aldrich, St. Louis, MO, USA) in ammonium acetate buffer (Sigma Aldrich, St. Louis, MO, USA), 0.4 M, pH 5 at 95 °C for 30 min. Free metal ions were eliminated via SepPak C-18 cartridge (Waters), preconditioned with methanol (Merck, Darmstadt, Germany) and water. The reaction mixture was loaded and the free ^nat^Lu was eluted with water while the metallated peptides were eluted with ethanol, evaporated to dryness, redissolved in water and lyophilized. The ^177^Lu-labeled conjugates were synthesized by dissolving 5–10 μg (3–6 nmol) of the DOTA-conjugates in 250 μL of sodium acetate buffer (0.4 M, pH 5.0) followed by incubation with [^177^Lu]LuCl_3_ (10–200 MBq, depending on the planned experiment) for 30 min at 95 °C. The stability of [^177^Lu]Lu-DOTA-ST8950 under two different storage conditions, room temperature (RT) and at 4 °C, was evaluated over 24 h after synthesis.

### 3.2. Log D Measurements

The determination of log *D* (pH = 7.4) was performed by the “shake-flask” method. To a pre-saturated mixture of 500 µL 1-octanol and 500 µL of phosphate-buffered saline (PBS) (pH 7.4), 10 µL of 1 µM of the ^177^Lu-labeled conjugates were added. The solution was vortexed for 1 h to reach the equilibrium and then centrifuged (3000 rpm) for 10 min. From each phase, 100 µL of the aliquot was removed and measured in a γ-counter. Each measurement was repeated three times. Care was taken to avoid cross-contamination between the phases. The partition coefficient was calculated as the average of the logarithmic values (*n* = 3) of the ratio between the radioactivity in the organic and the PBS phase.

### 3.3. Affinity Studies

The binding affinities of the ^nat^Lu-DOTA-ST8950, in comparison to ^nat^Lu-DOTA-NOC, were determined on membranes of HEK-SST2 and HEK-SST5 cells. The natural hormone Somatostatin-14 (SS-14) was used as reference compounds. ^125^I-labeled SS-14 (^125^I-SS-14, 81.4 TBq/mmol, Perkin Elmer, Waltham, MA, USA) was used as a radioligand for the competition binding assays. Binding assays were performed as described previously [27].

### 3.4. In Vitro Characterization of [^177^Lu]Lu-DOTA-ST8950

For all cell experiments, HEK-SST2 and HEK-SST5 were seeded at a density of 1 × 10^6^ cells/well in 6-well plates and incubated overnight with Dulbecco’s modified Eagle’s medium (DMEM) with 1% Fetal Bovine Serum (FBS, Biochrom GmbH, Merck Millipore, Darmstadt, Germany) to obtain a good cell adherence. For plating HEK-SST2 and HEK-SST5, the plates were pre-treated with a solution of 10% poly-lysine to promote the cell attachment.

#### 3.4.1. Internalization Assay

The cells were washed with PBS and were incubated with fresh medium (DMEM with 1% FBS) for 1 h at 37 °C. [^177^Lu]Lu-DOTA-ST8950 and [^177^Lu]Lu-DOTA-NOC (100 μL, 2.5 nM) were added to the medium (0.9 mL) and the cells incubated (in triplicates) for 0.5, 1, 2 and 4 h at 37 °C, 5% CO_2_. The internalization was stopped by removing the medium and washing the cells with ice-cold PBS. The cells were then treated twice for 5 min with ice-cold glycine solution (0.05 mol/L, pH 2.8), to distinguish between cell-surface-bound (acid releasable) and internalized (acid resistant) radio-peptide. Finally, the cells were detached with 1 M NaOH at 37 °C. To determine nonspecific cellular uptake, selected wells were incubated with the radio-peptide in the presence of 1000-fold excess of SS-14. Internalization and bound rate are expressed as a percentage of the applied radioactivity.

#### 3.4.2. Cellular Retention (Efflux) Assay

For the cellular retention studies, HEK-SST2 cells were incubated with both radio-peptides (2.5 nM) for 120 min. The medium was then removed and the wells were washed twice with 1 mL ice-cold PBS. The acid wash with a glycine buffer of pH 2.8 was performed twice (5 min each time) on ice to remove the receptor-bound radio-peptide. Cells were then incubated again at 37 °C with fresh buffer (DMEM with 1% FBS). After preselected time points (10, 20, 30, 60, 120 and 210 min) the external medium was removed for quantification of radioactivity and replaced with fresh 37 °C medium. The cells were solubilized in 1 M NaOH and collected for quantification.

### 3.5. In Vivo Evaluation of [^177^Lu]Lu-DOTA-ST8950 in Tumor-Bearing Mice

#### 3.5.1. Tumor Implantation

The Veterinary Office (Department of Health) of the Cantonal Basel-Stadt approved the animal experiments (approval no. 2799) in accordance with the Swiss regulations for animal treatment. Female athymic Nude-*Foxn1*^nu^/*Foxn1*^+^ mice (Envigo, The Netherlands), 4–6 weeks old, were injected subcutaneously with 10^7^ HEK-SST2 cells in the right shoulder and 10^7^ HEK-SST5 cells in the left shoulder, freshly suspended in 100 μL sterile phosphate-buffered saline. The tumors were allowed to grow for 2–3 weeks.

#### 3.5.2. Investigation of the Influence of the Injected Peptide Mass

Groups of mice bearing dual SST2- and SST5-expressing xenografts were injected with two different peptide doses of [^177^Lu]Lu-DOTA-ST8950: 10 pmol/100 µL/0.5–0.6 MBq and 100 pmol/100 µL/0.6–1.5 MBq. The biodistribution was evaluated at 1 and 4 h post injection. At the selected time points, the mice were sacrificed and the organs of interest were collected, rinsed, blotted, weighed and counted in a γ-counter. The results are expressed as a percentage of injected activity per gram (%IA/g) obtained by extrapolation from counts of an aliquot taken from the injected solution as a standard.

#### 3.5.3. In Vivo Metabolic Stability Studies

The in vivo stability of [^177^Lu]Lu-DOTA-ST8950 was evaluated after intravenous injection of (100 pmol/100 µL/9.3 MBq) into the tail vein of non-tumor-bearing mice. Blood samples were collected at 30 and 60 min after injection in polypropylene tubes containing ethylenediaminetetraacetic acid (EDTA). After centrifugation at 4 °C, the plasma was collected and 95% ethanol was added in equal volume (1:1 *v*/*v*). The mixture was stirred, centrifuged and the supernatant was separated from the precipitated proteins. The solution was treated with acetonitrile in equal volume (1:1, *v*/*v*) to promote further precipitation of proteins. After centrifugation, the supernatant was filtered, diluted with water (1:1, *v*/*v*) and analyzed by radio-RP-HPLC for the identification and quantification of the intact peptide and possible metabolites.

#### 3.5.4. Specificity and Kidney Protection

The in vivo SST2- and SST5-mediated uptake of [^177^Lu]Lu-DOTA-ST8950 was assessed in mice bearing HEK-SST(−) negative xenografts after injection of 100 pmol/100 μL/0.3 MBq. The mice were sacrificed 4 h p.i. and accumulation of the radiotracer in the tumors and in all organs of interest was quantified in a γ-counter.

The basic amino acid lysine was evaluated as a nephroprotective agent, in an attempt to reduce the kidney uptake of [^177^Lu]Lu-DOTA-ST8950. Dual SST2- and SST5-expressing xenografts were treated intravenously with lysine (20 mg/100 μL in PBS) 5 min before the administration of [^177^Lu]Lu-DOTA-ST8950 (100 pmol/100 μL/0.3 MBq). Animals were sacrificed 4 h p.i. and accumulation of the radiotracer in the tumors and in all organs of interest was quantified in a γ-counter.

#### 3.5.5. Pharmacokinetics of [^177^Lu]Lu-DOTA-ST8950

Quantitative biodistribution studies of [^177^Lu]Lu-DOTA-ST8950 (100 pmol/100 μL/0.6–1.5 MBq) was followed from 1 up to 168 h p.i. At the preselected time points, the mice were sacrificed and the organs of interest were collected, rinsed, blotted, weighed and counted in a γ-counter. The results are expressed as a percentage of injected activity per gram (%IA/g) obtained by extrapolation from counts of an aliquot taken from the injected solution as a standard.

#### 3.5.6. SPECT/CT Imaging

Mice bearing dual SST2- and SST5-expressing xenografts were imaged using a nano SPECT/CT system (Bioscan, Mediso, Budapest, Hungary) 4 h after administration of [^177^Lu]Lu-DOTA-ST8950 (100 pmol/100 µL/6 MBq). A helical CT scan was acquired with the following parameters: current, 177 mA; voltage, 45 kVp; pitch, A helical SPECT scan was acquired using multipurpose pinhole collimators (APT1), 20% energy window width centered symmetrically over the 208 and 113 keV g-peaks of ^177^Lu, 24 projections, and 1200 s per projection. CT and SPECT images were reconstructed and filtered using the manufacturer’s algorithm, resulting in a pixel size of 0.3 mm for the SPECT and of 0.2 mm for the CT.

#### 3.5.7. Dosimetry

Mice biodistribution data were used to generate time–activity curves for each radiotracer. Because of the absence of a specific radioactivity accumulation in bones and red marrow, a linear relationship between the blood and the red marrow residence times was assumed for estimating the red marrow radiation dose [40]. The proportionality factor was the ratio between the red marrow mass and the blood mass in humans. OLINDA/EXM 1.0 (OLINDA/EXM^®^, Vanderbilt University, USA) was used to integrate the fitted time–activity curves and to estimate the organ and effective doses using the whole-body adult female model. For all calculations, the assumption was made that the mouse biodistribution, determined as the %IA/organ, was the same as the human biodistribution.

### 3.6. Statistics

Comparison of data was performed using unpaired two-tailed *t*-test with GraphPad Prism 7 software (GraphPad Software, Inc., San Diego, CA, USA). *p* values < 0.05 were considered significant.

## 4. Conclusions

The 2-iodo-substituted somatostatin analog [^177^Lu]Lu-DOTA-ST8950 has an affinity for the SST2 and SST5, similarly to [^177^Lu]Lu-DOTA-NOC. The in vivo uptake and residence time of [^177^Lu]Lu-DOTA-ST8950 is high on SST2-expressing tumors, but significantly lower on SST5-expressing tumors. Nevertheless, SST5-targeting may provide additive value in the case of SST2 and SST5 coexpression in the same tumor, like gastroenteropancreatic neuroendocrine tumors, pituitary tumors and gastric cancers. [^177^Lu]Lu-DOTA-ST8950 has an effective dose similar to [^177^Lu]Lu-DOTA-TATE, however, its persistent kidney uptake is a drawback since nephrotoxicity is of concern in targeted radionuclide therapy with radiolabeled somatostatin analogs.

## Figures and Tables

**Table 1:** Analytical data of the DOTA-conjugates and of their corresponding ^nat^Lu complexes.

Compounds	Purity (%)	MW (Calculated)	MW (Observed)	HPLC (t_r_ min)
DOTA-ST8950	100	1699.5	1700.1	10.51
DOTA-NOC	96	1454.6	1456.2	11.00
^nat^Lu-DOTA-ST8950	100	1870.5	1870.8	11.03
^nat^Lu-DOTA-NOC	97	1625.6	1627.3	11.52

MW: molecular weight; t_r_: retention time.

**Table 2:** Binding affinities of the ^nat^Lu-DOTA-conjugates for somatostatin receptor subtype 2 (SST2) and SST5, compared to reference compounds.

	SST2		SST5	
Compounds	IC_50_ (nM)	95% Confidence Intervals (nM)	IC_50_ (nM)	95% Confidence Intervals (nM)
Somatostatin-14 ^¥,^*	0.11	0.08 to 0.15	0.35	0.22 to 0.55
ST8950 ^¥^	0.28	0.19 to 0.42	0.77	0.48 to 1.2
^nat^Lu-DOTA-ST8950	0.37	0.22 to 0.65	3.4	2.3 to 5.2
^nat^Lu-DOTA-NOC	0.51	0.33 to 0.78	4.8	3.1 to 7.6
^nat^Ga-DOTA-ST8950 ^¥^	0.32	0.20 to 0.50	1.9	1.1 to 3.1
^nat^Ga-DOTA-NOC ^¥^	0.70	0.50 to 0.96	3.4	1.8 to 6.2

IC_50_: half maximal inhibitory concentration; Experiments were performed in 3 to 4 separate sessions in duplicate; * somatostatin-14 is the natural ligand and was used as control; ^¥^ from [29].

**Table 3:** Biodistribution results of 10 vs. 100 pmol [^177^Lu]Lu-DOTA-ST8950 on dual SST2- and SST5-expressing xenografts at 1 and 4 h p.i. The results are expressed as mean of the % injected activity per gram of tissue (%IA/g) ± standard deviation (SD).

	1 h			4 h		
Organ	10 pmol *	100 pmol ^&^	*p*	10 pmol *	100 pmol ^#^	*p*
Blood	1.30 ± 0.24	0.96 ± 0.23	0.034	0.15 ± 0.02	0.09 ± 0.03	<0.001
Heart	0.84 ± 0.18	0.69 ± 0.09	*0.075*	0.20 ± 0.04	0.17 ± 0.03	*0.140*
Lung	7.12 ± 2.59	3.90 ± 0.84	0.011	3.81 ± 0.98	1.69 ± 0.40	<0.001
Liver	2.73 ± 0.59	2.60 ± 0.42	*0.660*	2.20 ± 0.27	2.19 ± 0.48	*0.998*
Pancreas	5.99 ± 1.75	6.36 ± 1.39	*0.693*	3.21 ± 0.50	4.38 ± 1.02	0.029
Spleen	1.82 ± 0.26	1.29 ± 0.19	0.002	0.96 ± 0.18	0.68 ± 0.11	0.001
Stomach	9.68 ± 2.34	5.63 ± 0.79	0.002	6.03 ± 1.28	4.94 ± 0.90	*0.063*
Intestine	3.08 ± 0.38	1.95 ± 0.65	0.006	2.29 ± 0.42	1.14 ± 0.42	<0.001
Adrenal	6.01 ± 1.15	4.23 ± 0.68	0.007	5.37 ± 1.17	5.21 ± 1.92	*0.952*
Kidney	11.72 ± 1.79	9.88 ± 0.99	0.045	10.84 ± 1.27	9.75 ± 1.87	*0.254*
Muscle	0.40 ± 0.07	0.30 ± 0.07	0.031	0.13 ± 0.02	0.10 ± 0.02	0.005
Bone	1.35 ± 0.25	0.74 ± 0.11	<0.001	1.12 ± 0.38	0.49 ± 0.11	<0.001
SST2-tumor	12.12 ± 3.94	14.17 ± 1.78	*0.322*	10.34 ± 2.78	15.50 ± 3.63	0.016
SST5-tumor	1.94 ± 0.46	1.78 ± 0.35	*0.561*	1.52 ± 0.75	1.87 ± 0.49	*0.240*

* *n* = 5, ^&^ *n* = 7; ^#^ *n* = 12; *p* < 0.05 statistically significant (black), *p* > 0.05 statistically not significant (italics).

**Table 4:** Biodistribution results of [^177^Lu]Lu-DOTA-ST8950, 4 h p.i. in SST-negative (SST(−)) tumor and after 5 min pre-injection of lysine (20 mg/100 µL) on dual SST2- and SST5-expressing xenografts. The results are expressed as mean of the % injected activity per gram of tissue (%IA/g) ± standard deviation (SD).

Organ	Control ^¶^	SST(−) xenograft ^¥^	Lysine ^§^
Blood	0.09 ± 0.03	0.10 ± 0.02	0.09 ± 0.02
Heart	0.17 ± 0.03	0.18 ± 0.03	0.18 ± 0.03
Lung	1.69 ± 0.40	2.43 ± 1.21	1.78 ± 0.67
Liver	2.19 ± 0.48	2.28 ± 0.20	2.25 ± 0.37
Pancreas	4.38 ± 1.02	n.d.	4.10 ± 1.29
Spleen	0.68 ± 0.11	0.84 ± 0.06	0.73 ± 0.17
Stomach	4.94 ± 0.90	4.89 ± 0.94	2.87 ± 0.63
Intestine	1.14 ± 0.42	1.35 ± 0.14	1.05 ± 0.37
Adrenal	5.21 ± 1.92	5.45 ± 2.26	4.87 ± 1.49
Kidney	9.75 ± 1.87	8.96 ± 1.39	5.93 ± 0.56
Muscle	0.10 ± 0.02	0.15 ± 0.02	0.12 ± 0.02
Femur	0.49 ± 0.11	0.56 ± 0.24	0.55 ± 0.16
SST2-tumor	15.50 ± 3.63	-	15.07 ± 2.32
SST5-tumor	1.87 ± 0.49	-	1.69 ± 0.52
SST(-)-tumor	-	0.70 ± 0.08	-

n.d. = not determined, ^¶^ *n* = 12 (see Table 3); ^¥^ *n* = 3; ^§^ *n* = 7.

**Table 5:** Results of the pharmacokinetics studies of [^177^Lu]Lu-DOTA-ST8950 (100 pmol) on dual SST2- and SST5-expressing xenografts. The results are expressed as mean of the % injected activity per gram of tissue (%IA/g) ± standard deviation (SD).

Organ	1 h *	4 h ^#^	24 h	72 h	168 h
Blood	0.96 ± 0.23	0.09 ± 0.03	0.02 ± 0.00	0.01 ± 0.00	0.00 ± 0.00
Heart	0.69 ± 0.09	0.17 ± 0.03	0.08 ± 0.01	0.06 ± 0.02	0.04 ± 0.01
Lung	3.90 ± 0.84	1.69 ± 0.40	1.16 ± 0.10	0.46 ± 0.08	0.23 ± 0.09
Liver	2.60 ± 0.42	2.19 ± 0.48	1.23 ± 0.16	0.85 ± 0.21	0.54 ± 0.09
Pancreas	6.36 ± 1.39	4.38 ± 1.02	1.68 ± 0.27	0.72 ± 0.14	0.37 ± 0.05
Spleen	1.29 ± 0.19	0.68 ± 0.11	0.36 ± 0.24	0.38 ± 0.12	0.27 ± 0.05
Stomach	5.63 ± 0.79	4.94 ± 0.90	2.61 ± 0.44	1.66 ± 0.49	0.99 ± 0.04
Intestine	1.95 ± 0.65	1.14 ± 0.42	0.51 ± 0.09	0.28 ± 0.06	0.12 ± 0.02
Adrenal	4.23 ± 0.68	5.21 ± 1.92	3.81 ± 1.08	2.86 ± 1.30	1.84 ± 0.49
Kidney	9.88 ± 0.99	9.75 ± 1.87	5.99 ± 0.41	3.20 ± 0.84	1.42 ± 0.38
Muscle	0.30 ± 0.07	0.10 ± 0.02	0.06 ± 0.00	0.04 ± 0.01	0.01 ± 0.00
Femur	0.74 ± 0.11	0.49 ± 0.11	0.30 ± 0.05	0.19 ± 0.05	0.11 ± 0.01
SST2-tumor	14.17 ± 1.78	15.50 ± 3.63	9.32 ± 2.02	4.22 ± 1.43	0.79 ± 0.13
SST5-tumor	1.78 ± 0.35	1.87 ± 0.49	0.88 ± 0.16	0.35 ± 0.08	0.08 ± 0.01

* *n* = 7, ^#^ *n* = 12, *n* = 4 for all other groups.

**Table 6:** Radiation dose estimation, extrapolated from mice to humans and expressed as Mean Absorbed Dose (mGy/MBq) for [^177^Lu]Lu-DOTA-ST8950. [^177^Lu]Lu-DOTA-TATE values estimated with the same methodology [32] are reported only for comparison.

Organ/tissue	[^177^Lu]Lu-DOTA-ST8950 (mGy/MBq)	[^177^Lu]Lu-DOTA-TATE (mGy/MBq)^¥^
Adrenals	2.46 × 10^−1^	2.37 × 10^−1^
Intestine	5.57 × 10^−2^	3.38 × 10^−1^
Stomach	7.55 × 10^−2^	1.27 × 10^−1^
Heart	6.06 × 10^−3^	6.44 × 10^−3^
Kidneys	5.44 × 10^−1^	2.13 × 10^−1^
Liver	9.16 × 10^−2^	1.16 × 10^−2^
Lungs	1.27 × 10^−2^	4.07 × 10^−2^
Muscle	5.43 × 10^−4^	6.94 × 10^−4^
Pancreas	2.95 × 10^−1^	3.10 × 10^−1^
Red marrow	1.59 × 10^−3^	1.25 × 10^−3^
Spleen	4.66 × 10^−2^	3.13 × 10^−2^
Total body	7.01 × 10^−3^	7.04 × 10^−3^
SST2-tumor	5.07 × 10^−1^	3.33 × 10^−1^
SST5-tumor	6.89 × 10^−2^	
Effective dose (mSv/MBq)	2.52 × 10^−2^	3.17 × 10^−2^

^¥^ The results of [^177^Lu]Lu-DOTA-TATE refer to the injected peptide mass of 10 pmol [32].
